# Valedictory Address

**Published:** 1871-09

**Authors:** W. C. Stanley


### VALEDICTORY ADDRESS.

BY W. C. STANLEY.

Bead before the Indiana State Dental Society.

Gentlemen of the Indiana State Dental Association:—The precedent has been established, and the practice maintained, which leads us to expect something by way of valedictory from the retiring president, before giving up to his successor in office, the post of honor.

Yet, in view of the fact that we have distinguished visitors with us, who have come here from love of the profession and a desire for its advancement, and to whom we are all anxious to listen—I would do injustice to my own feelings and to you, did I continue my remarks to any considerable length at this time.

It has rejoiced me to meet once again with you—to see the kindly smile of recognition of those who have so often met before on such occasions as this, and to feel the warm grasp of the hand of those who never knew the feeling of professional jealousy, but who meet every one who has chosen for his life work, the profession we love so well—and is seeking with earnest purpose to fulfill his mission faithfully, as a brother, and bid him God speed on his way.

Twelve years ago, I was present at the organization of this association, then a student of but a few months, and nothing but sickness has ever kept me from being present at its annual meetings.

At our second meeting, our esteemed friend, Dr. Knapp, was chosen chairman, and I well recollect with what warmth and earnestness in his valedictory the succeeding year, he urged upon us the necessity of making thorough preparation for the work before us.

I had, I confess, chosen this calling without any just conception of its responsibility or its capability. I did not know there was such a thing as a dental college in existence, I knew it had some literature, but not its extent. Nor was I alone in this ignorance. After my determination to become a dentist was fixed, I confided my plans out of defference, to our family physician, a man who stands now and stood then, second to but few in his own profession in our State. He sought to discourage me from my purpose, and from his remarks, I feel sure that he was as entirely ignorant of the fact that there was a *dental* profession in existence as myself. And though I had made *some* advance before first attending the association, the spirit exhibited, the earnest words of counsel and encouragement given, then opened a new field of thought to me, and gave me enlarged views of the character and dignity of my chosen profession. And however far I may have fallen short of fulfilling worthily its responsible duty—what little of success I may claim, I attribute in no small degree to the spirit infused on that and similar occasions.

In looking over the progress of dentistry for the last decade, we find much to commend—much to encourage us to persevere in our efforts towards perfection; yet, we regret to be compelled to admit that the progress falls far short of what it should be. Quackery and empiricism are yet abroad in the land, and seem to have secured a new lease of life, for they have never shown a more unblushing front than now. And the question arises, How shall we hold them in check and protect the people from their influence?

Legal enactments are generally conceded to be the *only* means for doing this—poisonous, noxious plants, thrive side by side with the useful and beautiful, and in the same soil, and experience has shown that to stimulate the growth of the good, does *not* eradicate the bad, but the principle of extermination is applied, and beauty and plenty are the result. So it is in professional life, side by side with our colleges, will thrive ignorance, as debasing quackery, as unblushing as can be found in any part of the country, and this must ever be the case so long as intelligence is not diffused among the masses, for being incapable of distinguishing the good from the bad, they go to those who promise most.

But how are we to secure legal protection? Our legislators will be slow to grant that for which so few ask, while the many are indifferent or opposed.

And this brings us to recognize the fact that we need to have identified with us, a larger numerical force—we need to draw into the current of progress a larger portion of the rank and file of the profession. And yet our accessions must necessarily come from the indifferent—from those who have heretofore kept aloof from us, but whose hearts are right, and who need but to be aroused to the consciousness that they are needed in the battle against the wrong, to bring them along with us.

It is an old truism that in union there is strength—this is only true when there is at least a minimum of strength in the individual members composing the union, and not otherwise. Hence, we see the necessity of guarding well the entrance to this association, that nothing may enter to mar the good work. But there are, I have no doubt, many good and true men who would give strength and influence to this association, who ought to be with us on this and all such occasions.

How shall we reach them? The solution has been hinted at by another than myself, but accords with my own view, and seems to me full of force and worthy of our consideration.

It is to send laborers into the field—men whose hearts are in the work, and who are qualified both to instruct and inspire, for such *only* need we send, whose duty it shall be to organize local societies by calling meetings of the profession in different localities, and while laboring to enlighten them in other duties, let them not fail to preach the gospel of professional charity and brotherly kindness. Thus, I think, the discordant element may be harmonized, and the jealousies which result from non-intercourse will be removed.

And these local societies, once established, can but result in good—for once getting a foretaste of the good things in store for these who truly seek the right, they will be loth to return to their old way, and year by year new members would come up from them, to this association, full of warm and earnest spirit, the result of personal contact with warm and earnest spirits, and will willingly lend a helping hand to help forward any good work—and thus, I doubt not, may be secured the legislation so much needed for the protection of both the profession and the public.

